# Geographical accessibility of medicines: a systematic literature review of pharmacy mapping

**DOI:** 10.1186/s40545-020-00291-7

**Published:** 2021-03-04

**Authors:** Cindrel Tharumia Jagadeesan, Veronika J. Wirtz

**Affiliations:** Department of Global Health, Boston University School of Public Health Crosstown, 3rd floor, 801 Massachusetts Avenue, Boston, MA 02118 USA

**Keywords:** Accessibility, Pharmaceutical, Spatial analysis, Medicines

## Abstract

**Background:**

Measuring access to medicines has often been limited to assessing availability and affordability, while little is known regarding other dimensions of access including geographical accessibility. Our study aims to provide a systematic review of literature on the accessibility of medicines by studying the geographical distribution of pharmacies using Spatial Analytical methods.

**Methods:**

As systematic review of scientific peer-reviewed literature between 2000 and 2018 was carried out using PubMed, Web of Science, Google Scholar, Google and the Preferred Reporting items for Systematic Reviews and Meta-Analyses (PRISMA). Data regarding pharmacy density, distance to pharmacies in relation of pharmacy to sociodemographic factors and pharmacy characteristics were extracted from studies that meet the inclusion criteria.

**Findings:**

Twenty papers fulfilled our inclusion criteria, of which only three were from middle income countries and rest from high-income economies. Pharmacy density per population was reported in 15 studies. Although geographical information was utilized in all studies, only 14 studies reported distance to pharmacies represented as Euclidean (straight line) distance. Disparities in accessibility was reported according to population income and rural or urban location. Seven studies described additional pharmacy characteristics including opening hours, presence of a pharmacist and delivery services.

**Conclusions:**

Geographical accessibility is a key dimension of access to medicines. Pharmacy density per population is a relevant indicator to assess geographical accessibility which should be complemented by an equity analysis using socio-demographic information and population perception of accessibility.

## Introduction

Medicines play an integral part of health system [1]. Target 3.8 of SDG 3 is: ‘to achieve universal health coverage, including financial risk protection, access to quality essential healthcare services and access to safe, effective, quality and affordable essential medicines and vaccines for all’ [2]. Access to medicines is essential to improve the health outcome and achieving universal health care coverage [1]. Lack of access to medicines can result in increased preventable morbidity and mortality, loss of economic income and increased poverty. One of the main access points of medicines are pharmacies and other private sector outlets [3]. Pharmacies not only provide medicines but often offer primary care services, advice and consultation regarding common ailments which helps to improve the overall health of the population [4].

Penchansky’s and Thomas’ [5] concept of access states availability, accessibility, accommodation, affordability and acceptability as the different dimensions of access. Measuring access has often been limited to two of these five dimensions, namely availability and affordability. For instance, Target 3.b of the SDGs is measured as the proportion of health facilities that have a core set of relevant essential medicines available and affordable on a sustainable basis [2]. However, equally important for achieving access is geographical accessibility, commonly measured as geographical distribution of pharmacies within a region and density of pharmacies per population [6]. This is also reflected by the fact that the geographical location of residency is a core dimension when measuring health equities [7].

With developing technologies, we can identify geographical distribution of pharmacies within a region, by mapping pharmacies using spatial methods to determine the areas of pharmacy deserts and the areas of high pharmacy density [6]. However, the literature on geographical distribution of pharmacies is sparse. An exception is the study of pharmacy density in Chicago and the relation between their distribution and socio-economic characteristics of the neighborhood [8]. Our study aims to provide a systematic review of literature on the evidences available for geographical distribution of pharmacies across countries and their relationship between pharmacy density and other sociodemographic factors. This study contributes to the existing body of knowledge of measuring geographical accessibility of medicines (Additional file 1).

## Methods

This study follows the Preferred Reporting Items for Systematic Reviews and Meta-Analyses (PRISMA) [9].

### Data collection

Original studies describing the geographical distribution of pharmacies were included. We included articles from all countries regardless of the funding source of the study. The review timeline was restricted to 18 years (2000–2018) and only studies published in English.

A search strategy that combines key words “geographic distribution”,” spatial analysis”,” maps” and “pharmacy” was applied to PubMed and Web of Science (see Appendix 1 for the search terms). Additionally, we did open Google Scholar searches using the combination of terms outlined in Appendix 1. Duplicated articles were removed. We excluded articles that discussed the access to medicines but had no mention regarding the geographical distribution of pharmacies. Geographical distribution of pharmacies represented in any format (e.g.—maps and graphical representation) were included. Both qualitative and quantitative studies were included. The full text papers were assessed against the inclusion criteria by one researcher and those identified as relevant was checked again by another researcher.

### Data extraction

Data from the included studies were then extracted into an excel database under the following headings: author, title, year and location of the study, objective of the study, type of pharmacy described- whether public or private, sampling and study design, pharmacy data collection source, pharmacy census validation, pharmacy density per 10,000 population, distance to pharmacies on relation to population, description of pharmacies based on urban/rural regions, ethnicity, socio-economic status and description of pharmacy characteristics. We classified study countries based on income according to World Bank Country classification [10]. If a study did not provide sufficient information to assess bias (e.g. no data on sampling and pharmacy data sources) it was excluded. Type of pharmacy was described as public if it was run by the government and private if it was run by an independent or chain pharmacy. Studies were classified as ‘national’ and ‘local’ depending on the region of data presented. If the data was presented for the entire country, we considered them as ‘national’ and if the data was presented for a state/city in a country, we considered it to be ‘local’. Data sources for pharmacies and population were identified and listed. We considered the pharmacy census to be validated if the data obtained were cross-checked by either visiting pharmacies in person or making phone call to certain pharmacy to check the accuracy of the data obtained from the sources. Ethnicity was described in some studies based on the population of pharmacy’s neighborhood. Socio-economic status was accounted for as described by each study using the countries deprivation indices and registries. Pharmacy characteristics such as hours of operation, presence of pharmacists, prescription medication delivery services, in-store medication availability and medicines price described were extracted and synthesized.

### Data analysis

While the units of pharmacy density varied across studies, we retrieved and converted them to per 10,000 population to compare results across studies. Distance to pharmacies were described as mentioned in original studies, due to different units in which they were reported aggregation and comparison between studies was not feasible. While some studies measured distance to pharmacies based on population, some studies measured distance between two pharmacies. Studies that further described differences in distribution of pharmacies according to urban–rural regions, ethnicity and socio-economic status was identified. Key emergent themes recurring across the data were analyzed and a narrative review was conducted.

## Results

Pubmed database search resulted in 3528 papers and Web of Science resulted in 2806 papers, which was narrowed to 4676 after removing duplicates. 176 articles were selected on the basis of title screen, 20 articles were identified based on the inclusion criteria. Only 3 of those studies are from lower middle- and upper middle-income countries (Brazil, India and South Africa) [6, 11, 12], the rest are from high income countries. Data from high income economies are available from countries namely New Zealand [13], England[14, 15], Scotland [16], Italy [17], Portugal[18], Canada [19, 20] and United States [8, 21–28]. Studies reported the geographical distribution of pharmacies, in terms of population density, rural and urban areas, characteristics and policy guidelines in the region.

The characteristics of studies included in the review are presented in Table 1. While the majority (n = 18) of the studies were observation, using census pharmacy data with geographical mapping, two studies [12, 16] did a cross-sectional survey to map the distribution of pharmacies in Scottish Highlands and India respectively. Except two studies from United States before 2010, most studies (n = 17) were conducted after 2010 making use of more developed geographical information systems. The term community pharmacies was employed in most of the papers. Table 1 stratifies the community pharmacies into public and private pharmacies.

**Table 1:** Study characteristics

Author	Title	Country	Year	Objective	Type of pharmacies
Ikram et al	Disparities in spatial accessibility of pharmacies in Baton Rouge, Louisiana	Baton Rouge, Louisiana, United States	2015	Examine the geographic variability in accessibility Identify possible “pharmacy deserts” in the study area	–
Emmerick et al	Farmácia Popular Program (FPP): changes in geographic accessibility of medicines during ten years of a medicine subsidy policy in Brazil	Brazil	2015	Describe historical stages of FPP Identify associated changes in geographic accessibility of medicines through FPP over time	Public and private
Qato et al	‘Pharmacy Deserts’ Are Prevalent In Chicago’s Predominantly Minority Communities, Raising Medication Access Concerns	Chicago, United States	2014	Examine whether access to pharmacies varied across Chicago communities based on their racial or ethnic composition	Community pharmacy defined as pharmacy licensed to dispense prescription to public
Todd et al	Access all areas? An area-level analysis of accessibility to general practice and community pharmacy services in England by urbanity and social deprivation	England	2015	Determine the percentage of population in England that has access to a GP within 20 min walk Compare accessibility of GP premises to that of a community pharmacy and how that varies by urbanity and social deprivation	–
Todd et al	Cutting care clusters: the creation of an inverse pharmacy care law? An area-level analysis exploring the clustering of community pharmacies in England	England	2018	Explore the clustering of community pharmacies in England and Determine the relationship between community pharmacy clustering, urbanity and deprivation	–
Lin et al	Access to Community Pharmacies by the Elderly in Illinois: a Geographic Information Systems Analysis	Illinois, United States	2004	Examine the current geographic accessibility to community pharmacies by the elderly in Illinois and Compare the accessibility in urban and rural areas	–
Domnich et al	Assessing spatial inequalities in accessing community pharmacies: a mixed geographically weighted approach	Liguria, Italy	2016	Explore the spatial accessibility to pharmacies in Liguria from the perspective of local characteristics Identify disparities and their sources	–
Padeiro et al	Geographical accessibility to community pharmacies by the elderly in metropolitan Lisbon	Lisbon-Metropolitan Area, Portugal	2017	Measure geographical pedestrian accessibility to community pharmacies by elderly people in the Lisbon	–
Casey et al	Pharmacv Services in Rural Areas: is the Problem Geographic Access or Financial Access?	Minnesota, South Dakota and North Dakota, US	2002	Analyze the extent to which problems with access to pharmacy services exist in rural areas of three states	Private
Amstislavski et al	Medication deserts: survey of neighborhood disparities in availability of prescription medications	New York, United States	2012	Characterize medication access at the community level	74% Independent 26% chain
Norris et al	Geographical access to community pharmacies in New Zealand	New Zealand	2014	Explore pharmacy numbers and location changes in NZ and assess its impact on people with poor access to pharmacy services	–
Law et al	The geographic accessibility of pharmacies in Nova Scotia	Nova Scotia, Canada	2013	Identify current geographic distribution of pharmacies in Nova Scotia Study the impact of closures have on the access to pharmacies	Private
Law et al	Geographic Accessibility of Community Pharmacies in Ontario	Ontario, Canada	2011	Study current state of geographic access to pharmacies and Simulate impact of possible closures	–
Pednekar et al	Mapping pharmacy deserts and determining accessibility to community pharmacy services for elderly enrolled in a State Pharmaceutical Assistance Program	Pennsylvania, United States	2018	Identify pharmacy deserts in Pennsylvania for elderly enrolled in a State Pharmaceutical Assistance Program	Private, Community pharmacy defined as pharmacy licensed to dispense prescription to public
Rushworth et al	A cross-sectional survey of the access of older people in the Scottish Highlands to general medical practices, community pharmacies and prescription medicines	Scottish highland	2017	Quantify issues of access to GPs, community pharmacies and prescribed medicine in older people	–
Chisholm Burns et al	Evaluation of racial and socioeconomic disparities in medication pricing and pharmacy access and services	Shelby county, Memphis, Tennessee, United States	2017	Determine if there are racial and socioeconomic disparities in community pharmacy access, drug pricing, and services	–
Ward et al	Assessing equity in the geographical distribution of community pharmacies in South Africa in preparation for a national health insurance scheme	South Africa	2013	Examine the ownership and geographical distribution of community pharmacies between 1994 and 2001	Private
Qato et al	Pharmacy Accessibility and Cost-Related Underuse of Prescription Medications in Low-Income Black and Hispanic Urban Communities	South Side Chicago, United States	2017	Examine the association between pharmacy accessibility, utilization and cost-related underuse of prescription medications among residents of predominantly low-income, Black and Hispanic urban communities	–
Sabde et al	Mapping private pharmacies and their characteristics in Ujjain district, Central India	Ujjain district, Madhya Pradesh, India	2011	Survey pharmacies and map them on GIS to study their location, rural–urban distribution and Study their relationship to roadways and healthcare providers	Private
Qato et al	The availability of pharmacies in the United States: 2007–2015	United States	2017	Examine the trends in availability of community pharmacies and pharmacy characteristics associated with access to prescription medicines	Public and Private

Table 2 outlines the methodology and results described in papers. Data reported were available both at national (n = 5) and local (n = 15) levels. While some studies (n = 11) obtained pharmacy data from the country’s department of health, studies from England used Fuse Geo-Health Care Access Database, studies from Canada used college of pharmacist’s data, two studies [12, 21] conducted cross-sectional surveys to identify pharmacies, two studies [16, 22] conducted surveys after obtaining data from a data-base. Ikram et al. [23] did not report the data source of pharmacy in their paper. Six studies conducted validation of pharmacy data and one study [24] used an internally validated database.

**Table 2:** Methodology and Results

Country	National	Local	Country Population Census	Pharmacy data collection source	Pharmacy Census Validation	Pharmacy density per 10,000 population	Distance to pharmacies in relation to pop	Urban/Rural	Ethnicity	Socio-economic status
National level data: upper-middle-income economies										
Brazil	X		X	Saude Legis—database containing legislation and Brazilian Ministry of Health	–	Medium/large municipality: 1.07, Small municipality: 2.15	–	–	–	Wealthy areas have a higher coverage
South Africa	X		X	Department of Health, SAPC- South African Pharmacy Council	–	0.61	–	Urban (least rural) province: 0.99, Most Rural province: 0.27	–	Most deprived districts: 0.11/10,000, In province: 0.34/10,000
National level data: high-income economies										
England	X		X	Fuse Geo-Health Care Access Database	–	–	89.2%—within 20 min walk	–	–	–
England	X		X	Geo-Healthcare Access Database	–	–	75%—within 10 min walking distance of one another 19%—in a cluster of two 56%—in a cluster of three	Urban: 19%, 19%, 62% Town: 94%, 5%, 1% Rural: 94%, 4%, 2%—no cluster, cluster of two and three respectively	–	–
New Zealand	X		X	Till 2005—New Zealand Gazette, the official government journal. 2005–2010: MedSafe, a division of Ministry of Health	–	2.2	In 2010: 86.5%—within 5 km In 2010: 99.8% within 25 km	Pharmacies centralize in areas of high population	–	Most deprived areas (according to NZDep) are more likely to live close to a pharmacy
United States	X		X	National Council for Prescription Drug Program, 2007–15	Internal validation by NCPDP	2.11	–	–	–	Highest quintiles had threefold more pharmacies than those in lowest quintile
Regional level data: lower-middle-income economies										
Ujjain district, Madhya Pradesh, India		X	X	Survey by identifying private pharmacies in the community, list from pharmacy association	X	2.8	78% within 50 m from a health care provider, 17% within 50–100 m from a health care provider	Urban: 5.83/10,000. Rural: 0.84 per 10,000	–	–
Regional level data: high-income economies										
Baton Rouge, Louisiana, United States		X	X	Not explained	–	2.5	86%—within 10 min, 96%—within 15 min, Average travel time: 8.11 min	–	African–American have better accessibility to pharmacy (7.64 min) than white (8.59 min)	Elderly have easy access to pharmacies compared to general population
Chicago, United States		X	X	Illinois Department of Financial and Professional Regulation for the period 2000–12	–	0.64/census tract	–	–	Pharmacy deseeds—clustered on the south and west side in segregated black and Hispanic community	–
Illinois, United States		X	X	Illinois Department of Professional Regulation	–	Urban: 1.27 Rural: 0.38	Average distance: 2.73 km, For elderly: 3.05 km. Entire population: within 32 km, 0.1% rural – travel more	–	–	–
Liguria, Italy		X	X	Italian Ministry of Health’s open dataset	–	3.8	Mean distance to nearest pharmacy: 6.8 km, 81.7% municipality-had one pharmacy	Provincial level: 3.6/10,000 Municipal level: 11.07/10,000	–	–
Lisbon, Portugal		X	X	National Health System database	–	13.35	88%—live within 800 m Elderly: 89.1% within 800 m Pedestrian distance: 61.2% within 10 min walk, 76.9% within 15 min walk	Poorly accessible areas seen not only in rural areas but also in norther urbanization corridor	–	–
Minnesota, North and South Dakota, United States		X	X	State Boards of Pharmacy, Surveys conducted with developed survey instrument	X	–	Average distance: Minnesota: 18 km, South Dakota: 29.3 km, North Dakota: 32 km	Population > 32 km from a pharmacy: Minnesota—0.4%, South Dakota—7.3%, North Dakota—4.3%	–	–
New York, United States		X	X	New York State registries, survey of selected pharmacy	X	High poverty regions: 5.1 Low poverty regions: 3.5	–	–	–	For each 10%—point increase in the number of households in poverty, odds of one or more prescription medications unavailability on the pharmacy shelves increases by 24%
Nova Scotia, Canada		X	X	Nova Scotia college of Pharmacists	–	12.24	42% within 800 m, 62.6% within 2 km and 78.8% within 5 km	Urban: 61.3%within 800 m, 90% within 2 km and 99.2% within 5 km. Rural:28% within 2 km and 53.3% live within 5 km	–	–
Ontario, Canada		X	X	Ontario College of Pharmacists	X	7.96 (From Nova Scotia article)	63.6%—within 800 m, 84.6% within 2 km and 90.7% within 5 km	Urban: 73.3%, 96.2% and 99.4% within 800 m, 2 km and 5 km respectively Rural: 8.5%, 18.1%, 40.9% within 800 m, 2 km and 5 km	–	–
Pennsylvania, United States		X	X	Pennsylvania Pharmaceutical Assistance Contract for the Elderly (PACE) and PACE Needs Enhancement Tier	X	–	33%—live more than 1.6 km	Pharmacy deserts – in rural	Pharmacy deserts have significantly more females, married and white elderly and fewer blacks and Hispanics compared to pharmacy non-deserts	Median annual household income higher for those living in pharmacy deserts
Scottish Highland, Scotland		X	CACL consumer database ‘Ocean’	Questionnaires sent to a random sample obtained from database ‘Ocean’. Pharmacy locations were identified using respondent’s postcode	–	–	Median distance travelled – 2.4 km 84.3%—convenient	Significant association between rurality and convenience – those in most rural areas more likely not convenient	–	Convenience, also associated with good health, younger age and those living with a partner
Shelby County, Memphis, Tennessee, United States		X	X	Cross-sectional survey of community pharmacies	X	1.49 ± 1.04	–	–	Areas with more pharmacies per 10,000 residents had a higher % of white residents	High income regions: ≥ 1.88 Low income regions: < 0.72
South side Chicago, United States		X	South Side Health and Vitality Statistics	Illinois Department of Financial and Professional Regulation, interviews with pharmacies	–	1.8 per mi^2^	Median distance: 1.93 km, 31% fill prescription from nearest pharmacy	–	–	–

With respect to population, all studies (n = 20) utilized the latest available country’s population census. Pharmacy density data was reported in 15 studies. While Qato et al. [25] reported the density of pharmacies per square mile and Qato et al. [8] reported the density of pharmacies per census tract, other studies (n = 13) reported data based on population. Comparatively high pharmacy density per 10,000 population in Lisbon [18], Nova Scotia [19] and Ontario [20] respectively.

Geographical Information System (e.g. Arc GIS Software) was used for any necessary geo-processing in all the studies [29]. However, distance to pharmacies was reported only in 14 studies. The rest of the studies [6, 11, 14, 15], [26], [27] did not report the pharmacy distance even while using GIS software. While majority studies reported distance to pharmacies based on population accessibility, the study from India [12] reported data based on pharmacy distance from health care providers, the study from the UK [8, 14] reported pharmacy distance between two pharmacies. Studies that measured the distance of pharmacies in relation to population represented the data in Euclidean distance (straight line distance) from the center of the measured radius containing the population. Hot spot analysis, which is a spatial analysis technique used to identify clusters of high and low value, was used by one study [28] to measure accurate distance of pharmacies using their address and population address.

Studies (n = 11) differentiated the pharmacy density based on urban and rural areas according to country census classification. All studies concluded urban population have better access to pharmacies and shorter distances to travel. The study conducted in Lisbon [18], Portugal, found pharmacy deserts even in parts of urbanized corridors of Lisbon.

Some studies [21, 23, 24, 28] (n = 4) differentiated pharmacy density based on ethnicity. Interestingly the reports from these studies contrast each other. While Ikram et al. [23] and Pednekar et al. [28] found pharmacy deserts in areas with higher white population, Qato et al. [25] and Chisholm-Burns et al. [21] reported pharmacy deserts in areas of segregated black and Hispanic community.

Studies (n = 8) also differentiated pharmacy densities based on their socio-economic status. The majority of studies identified pharmacy deserts among population of lower socio-economic status. Conversely, two studies [13, 28] identified higher income communities within pharmacy deserts.

Several studies [12, 14, 21, 22, 25, 27, 28] described pharmacy characteristics along with accessibility. Interestingly, two of them [21, 27] also surveyed the pricing information of medications and reported the data. Pharmacy characteristics described are hours of operation, pharmacists available, prescription medication delivery services, in-store medication availability and affordability. Appendix 3 describes the details of pharmacy characteristics.

Four studies [21, 22, 27, 28] described hours of operation in the pharmacies. They reported that the least populated area typically reported fewer hours per week. Although the study from India [12] and one study conducted in the US [21] described the availability of pharmacists in a pharmacy, the findings related to socioeconomic level of the population were not significant to arrive at a conclusion. Three studies from the US [21, 22, 28] reported data on prescription delivery services. It is important to note that all articles were representing different regions in United States and such services were not described in any middle-income economies. Two of these studies [12, 28] also described details on medication availability. While Sabde et al. [12] identified no difference in availability in urban and rural pharmacies, Pednekar et al. [28] reported 15% of pharmacies had at least one item out of stock. Two studies [21, 27] in the US collected data on affordability. However, it was hard to synthesize the data across studies due to different methods of measuring prices.

### Discussion

This systematic review fills an important gap in our knowledge on geographical accessibility of pharmacies and contributes to develop measures for access to medicine. While the majority of the studies are from high income economies, pharmacy accessibility is particularly relevant in low- and middle- income countries since transportation can be expensive and difficult to access. The absence of studies from low- and middle-income countries could possibly be attributed to the challenges of maintaining an up-to-date pharmacy census with limited resources in these nations. The study team in India [12] addressed the challenge of the lack of a census by walking through a geographically defined area and identifying pharmacies, whichis a resource intensive method.

The majority of the studies measured the distance based on the ‘centroid’ approach, which considers the center of the geospatial unit (e.g., zip code, census block) and measures the straight line (also known as Euclidean distance) distance to the nearest pharmacy. As outlined by Pednekar et al. [28], this may lead to errors in measurement as the actual distance required to travel to pharmacy might be less or more depending on the geography of the surrounding land. Although Ikram et al. [23] explains that distance measured in ArcGIS software is an underestimate compared to Google Maps and the data can be correlated, with improving access to Google Maps technology and hot spot analysis, we recommend further studies to determine the actual distance instead of using the centroid approach. Some studies measure pharmacy distance to assess density in terms of population and other studies measure distance between two pharmacies. Standardized measure of representation of pharmacy density such as pharmacy per 10,000 people would allow for uniformity and comparability.

Perception is another important factor to assess accessibility. However, only one study analyzed perception of distance. Rushworth et al. [16] found that those in the most remote areas and those who prescribed five or more regular medicines were more likely to report inconvenience of access to prescribed medicines. While only 63.7% found traveling to pharmacy to be easy, 84.3% found access to pharmacies convenient. This shows that people’s perception of easy access to pharmacies also depends on other factors like number of prescribed medicines, mobility and reliance on others.

Finally, it would be important to correlate the different access dimensions including geographical distribution to measure access to medicines more comprehensively. As mentioned by Penchansky and Thomas, access is multi-dimensional. Higher density of pharmacy is found in urban areas and increased pharmacy density is associated with increased access to medicines. High income areas were noted to have increased access to pharmacies except for two studies [13, 28], which noted pharmacy deserts in higher median income regions. Amitslavski et al. [27] found significant difference and very limited stock and hours of operation of pharmacies in poor communities compared to those of wealthier communities. They also explained that higher odds of common medications being out of stock in poorer communities might be due to high poverty and low rates of prescription insurance coverage. Home medication delivery services, as mentioned by Chisholm-Burns et al. [21], would be a feasible option to explore in areas where access is limited. Overall, access to pharmacy appears sparse in low income and rural regions and measures to expand pharmacy access based on actual distance in those populations would be beneficial.

### Limitations

Literature searches were restricted to PubMed, Web of Science and Google Scholar and did not use other search engines. Although comprehensive search terminology was used, there might be some articles that were missed. Literature was searched only in English language. While attempts to draw conclusion was made with regards to the distance and geographical distribution of pharmacies in various countries, heterogeneity in reporting on this measure reduced the ability to summarize trends and establish patterns. Studies were conducted in different countries with differences in cultures and health care system which affects consumer perception.

## Conclusion

Geographical accessibility of pharmacies is one of the key dimensions of access to medicines. Disparities between rural and urban populations is an important challenge. The literature is scarce on studies assessing accessibility of medicines in particular in low- and middle- income countries. Expanding our knowledge on geographical access of pharmacies will enable us to provide better access to medicines, moving a step closer towards providing universal health coverage.

## Supplementary Information

**Additional file 1.** PRISMA Flow Diagram on Review Process.

## Data Availability

All data for analysis in this review is in the public domain.

## Appendix 1: Search terms

GIS (AND) access (AND) medicines.

GIS (AND) Pharmacies.

Mapping (AND) medicines (AND) access (AND) Low- and middle-income countries.

Mapping (AND) pharmacies (AND) Low- and middle-income countries.

Mapping (AND) access (AND) medicines.

Mapping (AND) pharmacies (AND) developing countries.

Mapping (AND) pharmacy deserts.

geographic distribution of pharmacies.

geographic mapping of pharmacies.

geographic distribution (AND) access to medicines (AND) developing countries.

medicine access (AND) developing countries.

spatial analysis (AND) pharmacies.

spatial analysis (AND) pharmacies (AND) Low and middle income countries.

spatial analysis (AND) access to medicines.

spatial analysis (AND) access (AND) pharmacies.

maps (AND) medicine access.

## Appendix 2: Inclusion criteria

- Article published between 2000–2019
- Available in PubMed, Web of Science and Google Scholar
- Should represent geographical distribution of pharmacies in any format – maps, graphical representation
- From any country
- Qualitative or quantitative studies

## Appendix 3: Pharmacy characteristics table

See Table

**Table 3:** Pharmacy characteristics table

Author	Type of pharmacy	Hours of operation	Pharmacists on staff	Prescription delivery services	Medication availability	Affordability	Other characteristics
Sabde et al	Surveyed only private pharmacies	–	Only 14% of surveyed pharmacies provided details. 11.58%—pharmacists that worked in 88% urban pharmacy	–	No difference in availability in urban and rural pharmacies	–	Infrastructure: Refrigerators availability: 73.39%—urban 20.4%—rural. Vaccine availability: 8.5%—urban 5.7%—rural
Casey et al	Independent: in one location—54.9% In more than one location: 12.7% Chain—28.9% Owned by hospital or clinic—3.2%	Mean: 57.0 h	1: 29.5% 2: 46.6% 3: 17.9% 4 or more: 6%	Private homes: 85.3% Nursing homes: 79%, Clinics: 39.5%, Other retail: 3.5%, Other: 22%	–	–	Relief coverage: Difficulty finding for vaccines: Very difficult: 55%, Very easy: 7.1%
Amitslavski et al	74%—Independent, 26%—chain	24 h pharmacies: 5%, open on Saturday: 91%, on Sunday: 47%, on both Saturday and Sunday: 45%	–	–	15%—one item out of stock 74.5%—5 or few items missing 25.5%—six or more items missing		–
Pednekar et al	Independent and chain pharmacies more likely in pharmacy non-deserts	24 h services: Pharmacy non-deserts: 1.2%, pharmacy deserts: 0.3%	–	Pharmacy deserts: 28.3%, Pharmacy non-deserts: 37%	–	–	–
Rushworth et al	–	–	–	–		–	Those prescribed five or more medicines have inconvenient access to pharmacies
Chisholm Burns et al	–	Pharmacies operated on Saturday and Sunday = 6.7%, Monday – Friday: 63.5% Pharmacies in the least populated area typically reported fewer hours per week than those in the most populated area	–	Mean: 27.1% Significant difference in the percentage of pharmacies offering home delivery and income, employment rate and crime risk score	–		Immunizations: 92.6%, Medication therapy management: 92.4%, Generic drug program: 85.4%
Qato et al	Chain: 69.2%, Independent pharmacy: 13.5%, Clinic/CHC: 17.3%	–	–	–	–	Cost related underuse: 10.3%, low income and uninsured residents traveled further	those with a greater number of prescriptions utilized pharmacy more: 68.1%

3
