# Supplementary material for: Geographical accessibility of medicines: a systematic literature review of pharmacy mapping
Source: J Pharm Policy Pract. 2021 Mar 4;14:28. doi: 10.1186/s40545-020-00291-7 (PMC7931596; doi:10.1186/s40545-020-00291-7)
Supplement: Supplementary file 1 — Additional file 1. PRISMA Flow Diagram on Review Process. [file 40545_2020_291_MOESM1_ESM.doc]

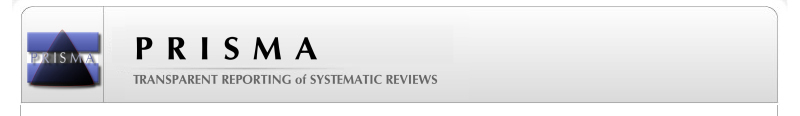
**Additional file 1: PRISMA 2009 Flow Diagram**

**Screening**

**Included**

**Eligibility**

**Identification**

Records identified through PubMed database
(n = 3528 )

Additional records identified through Web of Science
(n = 2806 )

Records after duplicates removed
(n = 4676 )

Records screened
(n = 4676 )

Records excluded
(n = 4500 )

Full-text articles assessed for eligibility
(n = 176 )

Full-text articles excluded, with reasons
(n = 156 )

Studies included in qualitative synthesis
(n = 20 )
